# Impact of Aging on the Phenotype of Invariant Natural Killer T Cells in Mouse Thymus

**DOI:** 10.3389/fimmu.2020.575764

**Published:** 2020-10-30

**Authors:** Georgia Papadogianni, Inga Ravens, Oliver Dittrich-Breiholz, Günter Bernhardt, Hristo Georgiev

**Affiliations:** ^1^Institute of Immunology, Hannover Medical School, Hannover, Germany; ^2^Research Core Unit Genomics, Hannover Medical School, Hannover, Germany

**Keywords:** invariant natural killer T cells, thymus, aging, transcriptome, IL2

## Abstract

Invariant natural killer T (iNKT) cells represent a subclass of T cells possessing a restricted repertoire of T cell receptors enabling them to recognize lipid derived ligands. iNKT cells are continuously generated in thymus and differentiate into three main subpopulations: iNKT1, iNKT2, and iNKT17 cells. We investigated the transcriptomes of these subsets comparing cells isolated from young adult (6–10 weeks old) and aged BALB/c mice (25–30 weeks of age) in order to identify genes subject to an age-related regulation of expression. These time points were selected to take into consideration the consequences of thymic involution that radically alter the existing micro-milieu. Significant differences were detected in the expression of histone genes affecting all iNKT subsets. Also the proliferative capacity of iNKT cells decreased substantially upon aging. Several genes were identified as possible candidates causing significant age-dependent changes in iNKT cell generation and/or function such as genes coding for granzyme A, ZO-1, EZH2, SOX4, IGF1 receptor, FLT4, and CD25. Moreover, we provide evidence that IL2 differentially affects homeostasis of iNKT subsets with iNKT17 cells engaging a unique mechanism to respond to IL2 by initiating a slow rate of proliferation.

## Introduction

Invariant natural killer T (iNKT) cells represent a subset of NKT cells that are distinguished by the expression of a very limited set of T cell receptors (TCR) (1). In mouse iNKT cells the Vα14Jα18 chain is always part of the TCR that recognize glycolipid ligands such as α-galactosylceramide (α-GalCer) presented to them in the context of CD1d, a non-polymorphic member of the MHCI-family (2, 3). In contrast to regular CD4 and CD8 T cells that undergo positive selection by thymic epithelial cells, iNKT cells are selected in thymus at the double positive (DP) stage by other DP thymocytes presenting endogenous glycolipid(s) (4, 5). Subsequent recruitment into the iNKT differentiation path is guided by a coordinated series of events such as upregulation of the transcription factors EGR2 and PLZF (6–8). Differentiation of murine iNKT cells was originally described as a stepwise process where cells pass through developmental stages S0 to S3 with cells at stage S3 (CD24^lo^CD44^hi^NK1.1^hi^) representing fully matured iNKT cells (9). iNKT cells are in general considered as phenotypically antigen-experienced cells placing them functionally at the interface between innate and adaptive immunity. A hallmark of iNKT cells is their capacity to quickly produce cytokines following stimulation by foreign antigen (10). Yet already in steady state iNKT cells produce messenger RNA (mRNA) coding for distinct cytokines (11, 12), and also secrete at least IL4 at physiologically relevant concentrations (13, 14). By investigating several gene deficient mouse models, evidence was provided that the available level of IL4 correlated positively with the generation of memory-like CD8 T (T_ML_) cells (13, 15, 16). Moreover, abundance of CD8 T_ML_ cells depends on age and mouse strain reflecting the frequency of IL4 producing iNKT cells in thymus (17). CD8 T_ML_ cells can emigrate from thymus (15) reinforcing the pool of peripheral virtual memory CD8 T cells that contribute to eliminate infections by distinct pathogens (18). It was shown that during differentiation (S1 to S3), iNKT cells convert gradually from IL4 to mainly IFNγ producers (19) but iNKT cells secreting IL17 discovered later on did not fit into this scheme easily (20). An alternative classification system was established that defines three main functional subsets of iNKT cells by correlating the expression pattern of the transcription factors PLZF, TBET, and RORγt, respectively, with a simple set of surface markers as well as the three key cytokines IFNγ, IL4, and IL17 (17, 21, 22): CD122^+^PLZF^lo^TBET^+^RORγt^−^ iNKT1 cells characterized by IFNγ secretion, CD122^−^CD4^+^PLZF^hi^TBET^−^RORγt^−^ iNKT2 cells producing mainly IL4 and CD122^−^CD4^−^PLZF^int^TBET^−^RORγt^+^ iNKT17 cells secreting predominantly IL17. Yet in agreement with the traditional classification system, the cytokine secretion patterns of iNKT subtypes are quite promiscuous (23).

It was observed earlier that there is a substantial shift in subset composition of iNKT cells in thymus of BALB/c and B6 mice when comparing young adults (6–10 weeks of age) with older animals (25–30 weeks of age) (15, 17, 24). This raised the question whether changes in gene expression occur over time and if so, whether this contributes to an age-dependent phenotype of iNKT cells. Based on a comparison of transcriptomes derived from young adult and aged iNKT subtypes we observed only minor differences on a global level but expression of some genes relevant for homeostasis and function of iNKT cells varied considerably between young and aged iNKT cells.

## Materials and Methods

### Animals

BALB/cAnNCrl (BALB/c) and C57BL/6N (B6) mice were bred in the animal facility of Hannover Medical School or purchased from Charles River Laboratories and kept under specific pathogen-free conditions. B6.129P2-*Blk^tm1Tara^*/J (*Blk*^−/−^) mice were purchased from Jackson Laboratory. All animals analyzed were female and 6–10 weeks old (young adults) or 25–30 weeks old (aged).

### *In Vivo* Labeling With Bromodeoxyuridine

Mice were injected intraperitoneally with 1.5 mg BrdU (Roche) on day 0 and gavaged with 5 mg BrdU on days 1–4. Mice were analyzed on day 5, the incorporated BrdU detected using a FITC BrdU Flow Kit (BD Pharmingen™).

### Cell Suspensions

Thymi, spleens, and peripheral lymph nodes were cut into small pieces, mashed through 40 μm cell strainers and washed once in FACS buffer (PBS with 3% FCS). For the isolation of lung lymphocytes, mice were perfused with cold PBS. Lungs were cut into small pieces and digested with 0.5 mg/ml collagenase D (Roche) and 0.025 mg/ml DNase I (Roche) in RPMI 1640/10% FCS for 30 min at 37°C. After digestion, EDTA was added at final concentration of 20mM and the samples were mashed through 40 μm cell strainers and washed once in RPMI 1640/10% FCS. Samples were next resuspended in 40% Percoll, layered on 70% Percoll and centrifuged for 20 min at 2,000rpm. Following centrifugation, the interphase containing the enriched lymphocytes was collected.

### Antibodies and Flow Cytometry

For extracellular staining, cells were blocked with rat or mouse serum 3% in FACS buffer and incubated with a-mouse antibodies for 30 min on ice. The following antibodies were used: anti-CD122 PE (TM-β1, 1:100), anti-CD122 biotin (TM-β1, 1:100), anti-CXCR3 PE (CXCR3-173, 1:100), anti-CD4 PerCP (RM4-5, 1:400), anti-CD4 BV510 (RM4-5, 1:200), anti-CD8α BV421 (53-6.7, 1:200), anti-CD8α APC/Cy7 (53-6.7, 1:200), anti-CD19 PE/Cy7 (6D5, 1:200), anti-B220 BV510 (RA3-6B2, 1:100), anti-IL17RB (9B10, 1:100), anti-CD3ε BV785 (145-2C11, 1:100), and streptavidin BV605 (1:400) all from BioLegend, anti-B220 eFluor450 (RA3-6B2, 1:200), anti-CD3e PE/Cy7 (145-2C11, 1:200) both from eBioscience, anti-CD25 Alexa 488 (PC61 5.3, 1:200) from Invitrogen, anti-CD4 BUV395 (GK1.5, 1:200), and anti-CD138 BV421 (281-2, 1:100) from BD Biosciences and rat IgG1 isotype control Alexa Fluor 488 (CAD9, homemade). CD1d tetramer loaded with PBS57 (analogue of a-GalCer) was provided by the tetramer facility of US National Institutes of Health and used in a dilution of 1:800.

For intracellular staining, cells were firstly incubated with zombie fixable viability dye (BioLegend) for 20 min at room temperature. Stainings were performed at room temperature using the Foxp3/transcription factor fixation/permeabilization buffer set or the intracellular fixation and permeabilization buffer set from eBioscience. The following antibodies were used: anti-IL4 BV421 (11B11, 1:100), anti-IFN-γ PE (XMG1.2, 1:100), anti-IL17A PE/Cy7 (TC11-18H10.1, 1:1,000), and anti-TBET PE/Dazzle 594 (4B10, 1:100) all from BioLegend, anti-granzyme A eFluor 450 (GzA-3G8.5, 1:100), mouse IgG2b kappa isotype control eFluor 450 (eBMG2b, 1:100), anti-PLZF Alexa Fluor 488 (Mags.21F7, 1:100), and anti-RORγτ PerCP-efluor710 (B2D, 1:600) from eBioscience and anti-RORγt PE/Cy7 (B2D, 1:800) from Invitrogen. Data were acquired on LSR II (BD) or Cytek Aurora and analyzed using FlowJo.

### *In Vitro* Culture of Invariant Natural Killer T cells

Thymic single cell suspensions were depleted of CD8^+^ cells (RmCD8-2, homemade) by using sheep anti-rat IgG Dynabeads (Invitrogen). Cells were then labelled with Cell Proliferation Dye eFluor 450 (eBioscience) and stained with appropriate antibodies. Total iNKT cells were sorted at the FACSAria Fusion (BD) as B220^−^CD8^−^CD3^+^CD1d tetramer^+^ cells with purity >90%. Sorted iNKT cells were then plated at a density of 15,000 cells/100μl RPMI 1640/10% FCS in a 96 well plate with or without recombinant IL2 (100U/ml) with or without anti-CD25 (PC61, 10μg/ml). Cells were then incubated for 3 days at 37°C.

### *In Vitro* Stimulation and Cytokine Production

For analysis of *Blk^−/−^* mice, total iNKT cells were sorted from splenic single cell suspensions as B220^−^CD3^+^CD1d tet^+^ cells with purity >90% and plated on a flat-bottom plate coated with anti-CD3 (clone 17A2, 1μg/ml) and anti-CD28 (clone 37.51, 2μg/ml). Brefeldin A was added at a concentration of 10μg/ml. Cells were incubated for 16 h at 37°C.

For analysis of cytokine production by aged thymocytes, total thymic single-cell suspensions from each mouse were depleted of CD8a expressing cells as described above. Next, depleted thymocytes were plated at a density of 2 x 10^6^/ml in RPMI 1640/10% FCS supplemented with PMA (phorbol 12-myristate 13-acetate; 50ng/ml), ionomycin (1.5µg/ml) and brefeldin A (10µg/ml). Plated cells were incubated for 3 h at 37°C. During this step control samples were kept on ice in FACS buffer. Following incubation cells were processed first for extracellular staining of cell markers followed by intracellular staining for detection of transcription factors and cytokines as described above.

### Isolation of RNA

Thymic cells were pooled from three female BALB/c mice, 6–7 weeks, or seven female BALB/c mice, 25–26 weeks old, respectively. iNKT cell subsets were sorted as iNKT1 (B220^−^CD3^+^CD1d tet^+^CD122^+^), iNKT2 (B220^−^ CD3^+^CD1d tet^+^CD122^−^CD4^+^) and iNKT17 (B220^−^ CD3^+^CD1d tet^+^CD122^−^CD4^−^). RNA was isolated using the RNeasyPlus Micro Kit (Qiagen).

### RNA-Sequencing and Raw Data Processing

#### Library Generation

1–5ng of total RNA was used for library preparation with the SMARTer Stranded Total RNA-Seq Kit v2—Pico Input Mammalian (Takara/Clontech) according to conditions recommended by the manufacturer. Generated libraries were barcoded with dual indexing approach and were finally amplified with 11 cycles of PCR. Fragment length distribution of generated libraries was monitored using Bioanalyzer High Sensitivity DNA Assay (Agilent Technologies). Quantification of libraries was performed by use of the Qubit^®^ dsDNA HS Assay Kit (Thermo Fisher Scientific).

#### Sequencing Run

Equal molar amounts of 13 libraries in total were pooled for a sequencing run. Accordingly, each analyzed library constitutes 7.7% of overall flowcell capacity. The combined library pool was denatured with NaOH and was finally diluted to 1.5 pM according to the Denature and Dilute Libraries Guide (Illumina). 1.3 ml of denatured pool was loaded on an Illumina NextSeq 550 sequencer using a High Output Flowcell for 75bp single reads (#FC-404-2005; Illumina).

#### Raw Data Processing and Quality Control

BCL files were converted to FASTQ files using bcl2fastq Conversion Software version v2.20.0.422 (Illumina).

The FASTQ files were adapter and quality trimmed using Trim Galore! (version 0.4.1) with default settings as described in the user guide except for the setting of the quality cutoff (-q/–quality) which was set to a Phred score of 15. Trim Galore! used Cutadapt (version 1.9.1) as subroutine.

Quality control of FASTQ files was performed by FastQC (version 0.11.4) before and after trimming.

#### Mapping

After trimming, FASTQ files were mapped against a reference genome with the splice-aware aligner STAR (version 2.5.0c) to generate BAM files. The BAM files were built in a two-pass Mapping (–twopassMode Basic) and were finally sorted (–outSAMtype BAM SortedByCoordinate). All other setting have been left as default as described in the manual. The genome index files were created by STAR with default settings using Mus musculus sequence and annotation data (UCSC, built mm10) available on Illumina’s iGenome site (http://support.illumina.com/sequencing/sequencing_software/igenome.html).

#### Quantification, Differential Expression Analysis

Generated BAM files were imported to our local Galaxy instance (release 17.05). Read counting was performed by featureCounts (Galaxy Tool Version 1.4.6.p5) with default settings except for “strand specificity of the protocol” which was set to “stranded (reverse)” and “minimum mapping quality per read” which was set to 10. Mus musculus annotation data (UCSC, build Mm10) were used.

Normalization and differential expression analysis was performed with DESeq2 (Galaxy Tool Version 2.11.39) with default settings except for “output normalized counts table” with was set to “yes.”

#### Generation of Excel Table Containing Normalized Count Data and Adjusted p-Values

DESeq2 was used to generate normalized read counts for all thirteen samples. Additional DESeq2 analyses were applied to retrieve adjusted p-values for the most relevant pairwise comparisons.

### Bioinformatics and Statistical Analysis

The normalized RNA reads were used to generate heat maps and volcano plots by using Qlucore Omics Explorer software. All statistical analyses were carried out using GraphPad Prism. Unpaired two-tailed t-test was used when analyzing two data sets while one-way ANOVA followed by Tukey’s multiple comparisons test were used when analyzing three or more data sets. Two-way ANOVA followed by Sidak’s multiple comparisons test were used when analyzing two different groups of data sets.

## Results

### Defining Thymic Invariant Natural Killer T Subsets of Different Ages

**Figure 1:**
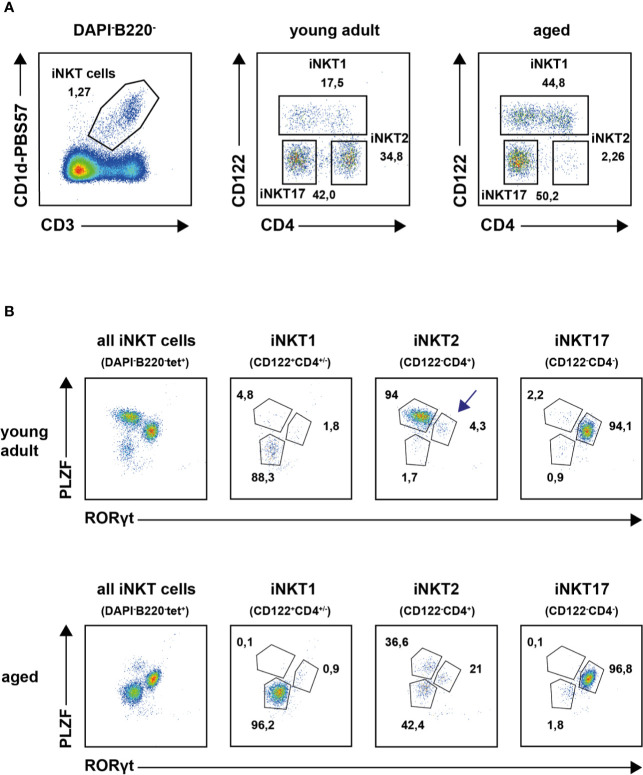
Thymic invariant natural killer T (iNKT) subsets in young adults and aged mice. **(A)** Representative plots illustrating thymic iNKT subset composition in young adult and aged BALB/c mice following application of the indicated gates. **(B)** Representative plot showing transcription factor staining of tet^+^ thymic iNKT cells (left panels) and of iNKT subsets as defined by surface markers CD122 and CD4 from young adult (upper panel) or aged (lower panel) BALB/c mice. Average frequencies are given for cells in the indicated gates derived from analysis of four young adult and five aged animals.

Recently, the expression signatures of iNKT1, 2 and 17 cells of young adult thymus of BALB/c and B6 mice were investigated in great detail (11, 12, 25). In this study, we were interested to decipher the effects of aging on the gene expression pattern of thymic iNKT cells by RNA sequencing. This required live cell sorting wherefore we intended to use surface markers CD122 and CD4 along with CD1d tetramer (tet) staining to obtain iNKT1, iNKT2 and iNKT17 cells (see *Introduction*, **Figure 1A**). In these stains we neglected CD24 expressing immature iNKT cells that comprise typically less than 1–2% of iNKT cells in thymus. To corroborate our sorting strategy, subtype phenotyping of young adult and aged iNKT cells was also done *via* detection of transcription factors PLZF and RORγt. In young adult mice the definition of tetramer (tet)^+^B220^−^DAPI^−^ iNKT subsets according to CD122/CD4 expression status (see **Figure 1A**, upper panel) correlated well with that of the transcription factors PLZF and RORγt confirming earlier observations (**Figure 1B**, upper panel) (12, 17). This was also observed for aged iNKT1 and iNKT17 cells but not for aged iNKT2 cells (**Figures 1A, B**, lower panels). Here, the CD122^−^CD4^+^ cells gave rise to three distinct subpopulations: PLZF^hi^RORγt^lo^ cells (presumably bona fide iNKT2 cells), PLZF^lo^RORγt^lo^ cells, and PLZF^int^RORγt^+^ cells, respectively. Because the numeric contribution of the latter two populations to the pool of aged iNKT2 cells is considerable, we addressed the aged CD122^−^CD4^+^ cells as iNKT2p (p = pool). Despite this limitation, two independent CD122/CD4 sorts each yielding cells from pooled thymi of young adult and aged mice were performed and subsequently their RNA was subjected to sequencing. All sequencing data were deposited in the GEO data base (accession number GSE147666). Re-analysis of cells immediately after sorting indicated that all collected populations were at least 95% pure except for aged iNKT2p cells that were contaminated by approximately 10% iNKT17 cells (**Supplementary Figure S1**). This was most likely due to the scarcity of iNKT2 cells in aged thymus. Apart from this, purity was also confirmed by the sequencing data because none to negligible counts were recorded for mRNAs typically expressed by dendritic cells (DC), macrophages, blood monocytes, NK cells, B cells, and diverse T cell subsets, respectively (e.g., *Itgax*, *Itgam*, *Csf1r*, *CD19*, *Fas*, *Foxp3*, *Ccr3*, *Ccr6*, *Il10*).

### Expression of Signature Genes in Young Adult and Aged Invariant Natural Killer T Subpopulations

**Figure 2:**
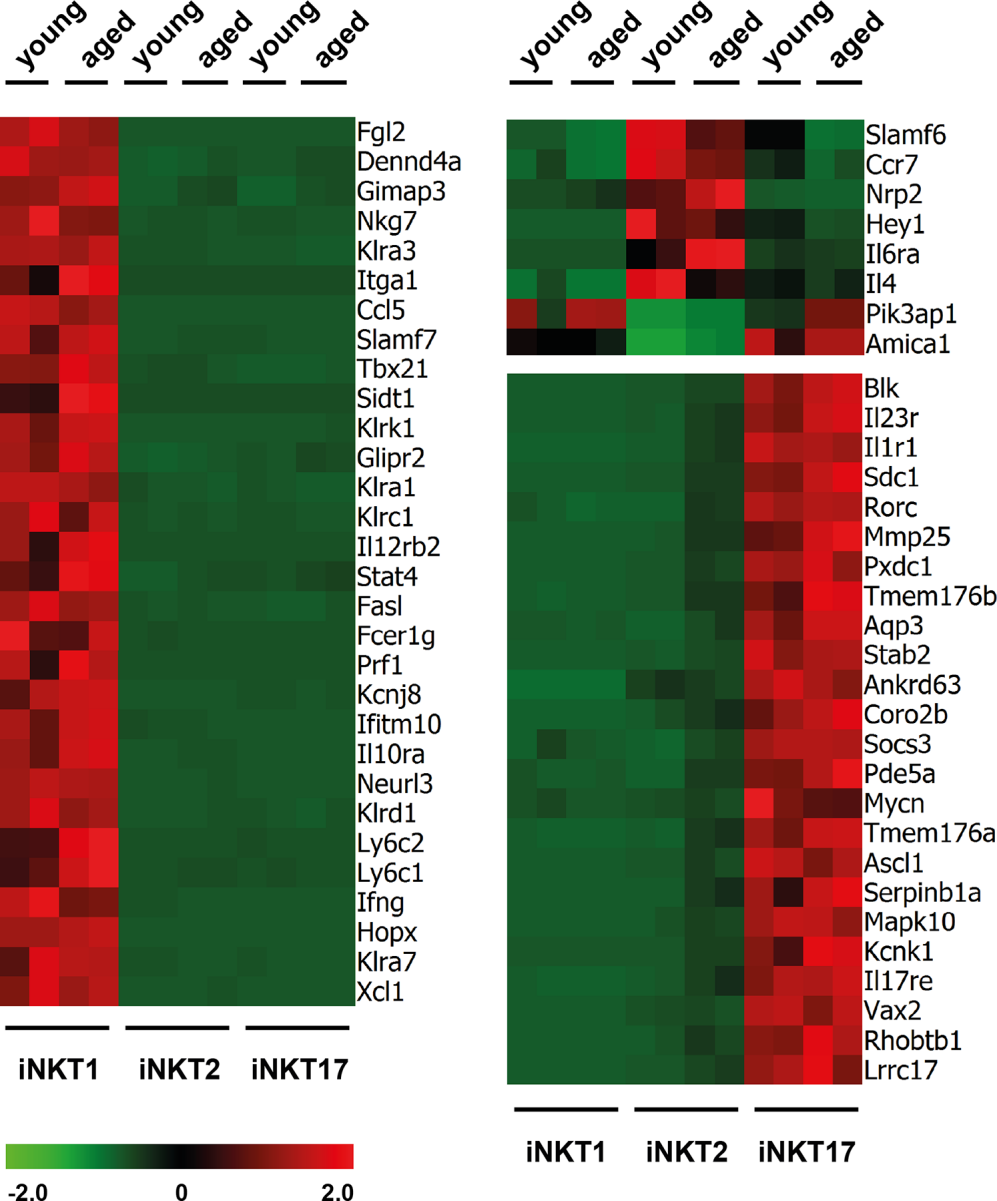
Expression of signature genes in invariant natural killer T (iNKT) subsets from young adults and aged mice. Heat maps depicting genes upregulated (red) or downregulated (green) in thymic iNKT1, 2 and 17 cells from young adult or aged BALB/c mice. Data from two independent experiments are shown as described in the text and *Materials and Methods*.

RNA sequencing revealed that the number of reads for signature genes specifying iNKT1 cells (*Tbx21*, *Fgl2, Klra3, Slamf7*, and others, see **Figure 2** and **Supplementary Table S1A**) were uniformly high in iNKT1 and very low for iNKT2 and iNKT17 cells in young adult as well as aged cells. Moderate age-related divergences in read counts were observed regarding some but not all signature genes yet these differences were less than 2.5 fold and for the most part statistically not significant.

Similarly, most iNKT17 signature genes were also expressed exclusively and uniformly high in young and aged iNKT17 cells (**Figure 2**) but their expression was also found to some extent in aged (but not young) iNKT2p cells reflecting the 10% sort impurity mentioned before (for example *Rorc*, *Sdc1*, *Il23r*, and others, **Supplementary Table S1A**). We confirmed earlier observations that *Blk* coding a tyrosine-protein kinase is expressed highly and specifically in iNKT17 cells therefore representing a novel iNKT17 marker (12). *Blk* was shown to be of importance for the generation of IL17 producing γδT cells (26) suggesting that BLK might also be important for iNKT17 cell biology. However, upon investigation of *Blk* deficient mice, we failed to detect any impact on the frequency of iNKT cells or their capacity to produce the key cytokines IFNγ, IL4, and IL17, respectively (**Supplementary Figure S2**).

**Figure 3:**
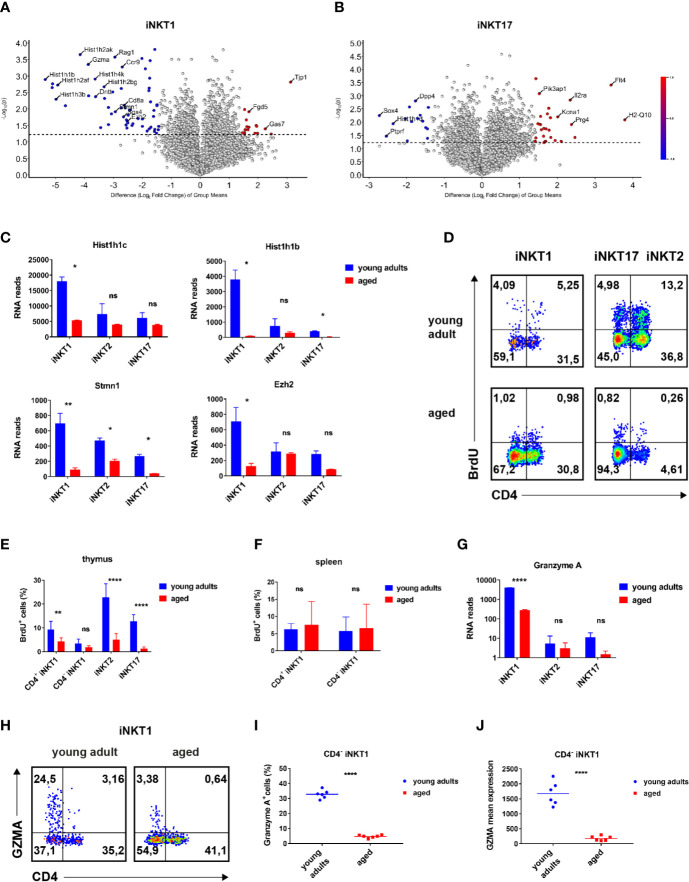
Age-dependent expressed genes iNKT1 and iNKT17 cells. **(A, B)** Volcano plots illustrating genes upregulated (red dots to the right) or downregulated (blue dots to the left) in thymic iNKT1 and iNKT17 cells from aged BALB/c mice when compared to young adults (fold change ≥2.83, p value <0.05; otherwise genes are represented by shaded dots). Data source as in **Figure 2**. **(C)** *Hist1h1c* and *Hist1h1b* RNA reads (mean ± sd) in thymic iNKT subsets. **(D–F)** BrdU detection at day 5 following BrdU administration *in vivo*. Gating strategy as indicated in **Figure 1A**. **(D)** Representative flow cytometry plots of BrdU levels in thymic iNKT subsets as indicated. **(E, F)** Summary of BrdU levels (mean ± sd) in thymic and splenic iNKT cells. Data were from two independent experiments with four young adult and four aged mice each. **(G)** *Gzma* RNA reads (mean ± sd) in the indicated thymic iNKT subsets. Data source as in **Figure 2**. **(H–J)** Analysis of granzyme A expression. Gating was done as shown in **Figure 1A**. **(H)** Representative flow cytometry plots showing granzyme A expression in thymic iNKT1 cells. **(I, J)** Granzyme A^+^ cell frequencies and mean expression among thymic CD4^−^ iNKT1 cells. Data were summarized from two independent experiments analyzing three young adult and three aged mice each. Two-way ANOVA followed by Sidak’s multiple comparisons test were performed in **(C)**, **(E, G)**. Unpaired t test was performed in **(I, J)**. *p < 0.05, **p < 0.01, ****p < 0.0001, ns, not significant.

**Figure 4:**
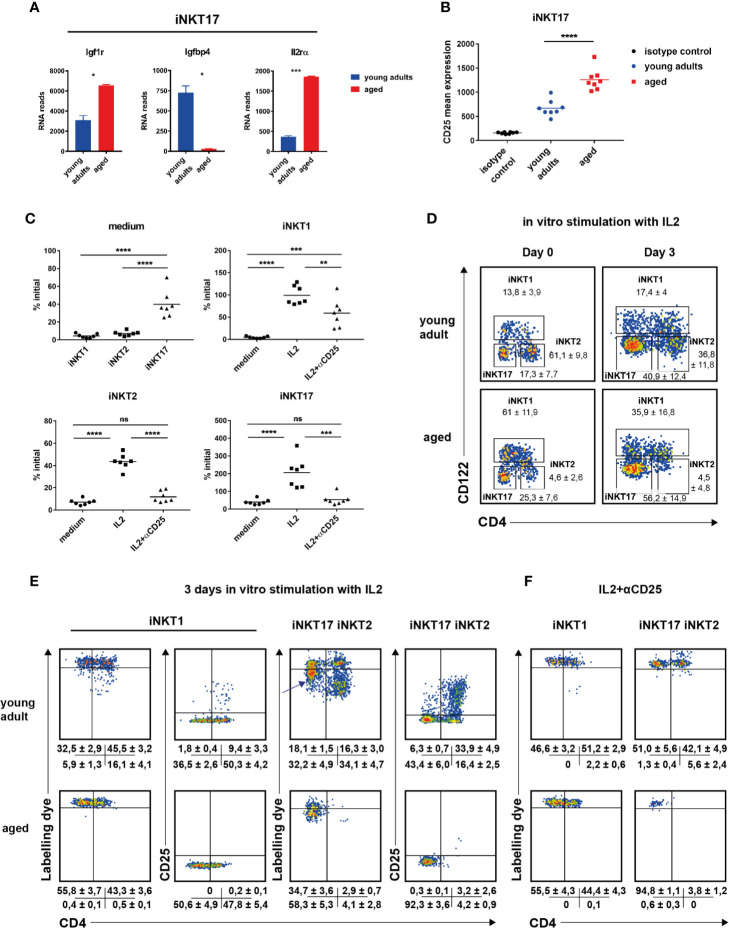
The role of IL2 in invariant natural killer T (iNKT) cell homeostasis. **(A)** *Igf1r*, *Igfbp4*, and *IL2ra* RNA reads (mean ± sd) in thymic iNKT17 cells from young adult and aged BALB/c mice. Data source as in **Figure 2**. **(B)** CD25 mean expression in thymic iNKT17 cells from young adult and aged BALB/c animals determined by flow cytometry. Gating as shown in **Figure 1A**. Data are collected from four independent experiments. Each dot represents data from one animal. **(C–F)** *In vitro* effects of recombinant IL2. Sorts and stains to obtain labeled iNKT cells were done as described in *Materials and Methods*. **(C)** Shown are % of initial cell numbers (day 0) on day 3 in medium or in the presence of IL2 ± anti CD25 antibody (αCD25). Data were collected from seven independent experiments (dots). **(D)** Representative flow cytometry plots of iNKT cells from young adult and aged BALB/c thymus on day 0 and day 3 after stimulation with IL2. Average frequencies (± SD) are given summarizing seven (aged) and eight (young adult) experiments. **(E, F)** Representative flow cytometry plots of the indicated iNKT subpopulations as gated in **(D)** showing distribution of cell tracker dye and CD25 levels on day 3 after stimulation with IL2 ± αCD25. Shown are the average frequencies for cells in the corresponding gates (± SD) underneath of each panel summarizing 5–7 independent experiments. Unpaired t test was performed in **(A)**. One-way ANOVA followed by Tukey’s multiple comparisons test were performed in **(B, C)**. *p < 0.05, **p < 0.01, ***p < 0.001, ****p < 0.0001, ns, not significant.

In agreement with earlier observations, the number of reliable signature genes specifying iNKT2 cells is much lower when compared to iNKT1 and iNKT17 cells (e.g., *Ccr7*, *Il6ra*, *Slamf6*, **Figure 2** and **Supplementary Table S1A**). Therefore, we also included in the heat map some genes that were expressed by iNKT1 and iNKT17 cells but not iNKT2 cells (e.g., *Pik3ap1*, **Figure 2**). Our data would also indicate that *Nrp2* (neuropilin 2) may serve as a new marker for iNKT2 cells. Neuropilin-2 was shown to be involved in diverse immune relevant activities (27). It is expressed by other T cell subsets as well as macrophages and DC. It was evident that the fluctuations in the read counts comparing expression of iNKT-specific genes in young adult *versus* aged cells was high in case of iNKT2p cells. This may be attributed to the heterogeneity of the aged iNKT2p cells but in essence, the iNKT2 signature genes used to demarcate young adult iNKT2 cells continued to define also aged iNKT2p cells (**Supplementary Table S1A**). However, based on their expression pattern regarding PLZF/RORγt, it appeared that the subpopulation of PLZF^lo^RORγt^lo^ cells represent iNKT1-like cells. Yet we failed to detect expression of typical iNKT1 signature genes among aged iNKT2p cells (**Supplementary Table S1A**). A parallel detection of several surface markers and transcription factors along with determination of the cytokine production pattern upon stimulation revealed that the PLZF^lo^RORγt^lo^ cells expressed TBET yet at a lower level than bona fide iNTK1 cells (**Supplementary Figure S3A**, blue histograms). In addition, these cells produce IFNγ upon stimulation with PMA/ionomycin (**Supplementary Figure S3B**, blue plots). Thus, the aged tet^+^CD122^−^CD4^+^PLZF^lo^RORγt^lo^ cells may represent precursor iNKT cells in a transitory stage. Alternatively, these cells may represent a separate iNKT cell population coming into existence preferentially in the environmental conditions of an aged thymus. Along this line, the tet^+^CD122^−^CD4^+^PLZF^int^RORγt^+^ cells do not represent bona fide iNKT17 cells. They may simply be iNKT17 variants that upregulated CD4 expression now being present in the CD122^−^CD4^+^ iNKT2 gate. Vice versa they could comprise more immature iNKT17 cells that develop into mature iNKT17 cells but still possess surface CD4. Possibly, this iNKT17-like population already exists among young CD122^−^CD4^+^ iNKT2 cells (see arrow in **Figure 1B**, upper panel) but is visible much better in older thymus when the number of bona fide iNKT2 cells decreased dramatically. Notably, we already detected earlier that a small fraction of iNKT2 cells sorted from young adult BALB/c thymus and stimulated *in vitro* with PMA/ionomycin expressed IL17, the key iNKT17 cytokine (12). The hypothesis that tet^+^CD122^−^CD4^+^PLZF^int^RORγt^+^ cells represent immature iNKT17 cells was corroborated by the finding that they are CD138^+^ and produce IL17 upon stimulation (**Supplementary Figures S3A, B**, red histograms and plots) while a larger proportion of them was IL17RB^+^ as compared to bona fide iNKT17 cells. Based on the intricacies regarding the nature and origin of the iNKT2p subpopulations, an unambiguous identification of age-related changes in the expression of iNKT2 specific genes appeared less plausible. Therefore, we restricted further analyses deciphering age-dependent expression of distinct genes to iNKT1 and iNKT17 cells. Nevertheless, analysis of iNKT1 and iNKT17 relevant genes necessitated including iNKT2 cells wherefore we provide data when appropriate (see **Figures 3** and **4** and list of genes presented in the **Supplementary Tables**).

### Genes Displaying an Age-Dependent Expression Pattern

In order to identify genes that are regulated in an age-dependent manner, we focused on genes that were expressed to a statistically significant and also to a substantially different degree in iNKT1 and iNKT17 cells depending on their age (volcano plots **Figures 3A, B**: >2.83 fold expression difference = 1.5 in log scale axis; significance level p < 0.05). Please note that for the sake of clarity, the list of exemplary genes in **Supplementary Table S1B** only encompasses genes with a >4 fold difference in expression between young adult and aged cells.

### iNKT1 Cells

A global survey of genes expressed by iNKT1 cells revealed that the number of significantly regulated genes is low (volcano plot, **Figure 3A**). However, a bias exists since more genes appear down-regulated whereas a prominent up-regulation upon aging was observed only for *Tjp1* (Tight Junction Protein ZO-1, **Figure 3A**; **Supplementary Table S1B**). It is unlikely that ZO-1 will enable iNKT1 cells to form classical tight junctions because the cells do not express genes coding for required accessory proteins like claudins (*Cldn1*, *Cldn2*) or occluding (*Ocln*) (28). ZO-1 may instead participate in the establishment of cytoskeleton-based structures like immune synapses. Altered cytoskeletal dynamics upon aging were also indicated by the down-modulation of *Stmn1* observed in all iNKT subtypes (**Figure 3C**). Stathmin was shown to regulate microtubule organization in activated T cells (29). Among the cluster of down-regulated genes many replication-dependent histone genes can be found (**Figure 3A**). Indeed, a more systematic check encompassing replication-dependent histone genes with highest read counts revealed that their expression was down-regulated in aged mice without any exception not only in iNKT1 but also in iNKT2p and iNKT17 cells (**Supplementary Table S2A**). However, the extent of down-regulation varies considerably. Whereas down-modulation was rather uniform and moderate in iNKT2p cells, the extent of regulation in iNKT1 cells is either comparable to that of iNKT2p cells (for example *Hist1h1c* and *Hist1h4d*) (30) or significantly more pronounced (for example *Hist1h1b*, **Figure 3C**; see also **Supplementary Table S2A**). In the latter cases, also in iNKT17 cells down-regulation was higher than average. In contrast, average expression of replication-independent histone genes was found to be decreased only moderately in all aged iNKT subtypes (**Supplementary Table S2B**). Interestingly, expression strength of replication-dependent histone genes in young adults obeyed a simple ranking: iNKT1>iNKT2>iNKT17 (**Supplementary Table S2A**). This is in contrast to the assumption that iNKT1 and iNKT17 cells are matured (i.e., rather non-dividing cells) whereas iNKT2 cells encompass more immature cells (17). Therefore, propagation of iNKT cells was investigated *in vivo* by bromodeoxyuridine (BrdU) incorporation (**Figures 3D–F**). In young adult thymus, iNKT2 cells divided more vividly than iNKT17 cells (**Figures 3D, E**). iNKT1 cells showed the lowest level of propagation yet CD4^+^iNKT1 cells incorporated BrdU to a larger extent than CD4^−^iNKT1 cells. Thus, the expression of RNA coding for replication-dependent histone genes is not reflecting the actual propensity of the corresponding iNKT subpopulations to propagate. Still, in accordance with the RNA sequencing data, the degree of cell division was substantially reduced in aged thymic iNKT cells. Remarkably, levels of BrdU incorporation into iNKT1 cells of spleen were slightly above 5% resembling those observed in thymus but were age-independent (**Figure 3F**, data for iNKT2 and 17 cells in spleen were too inconsistent due to the low frequencies of these cells). In all, the BrdU data shown here fit to the current view that iNKT1 cells are mostly quiescent whereas iNKT2 cells encompass the largest fraction of cycling cells with the iNKT17 cells somewhere in between (11).

Among granzyme family members, only *Gzma* and *Gzmb* were found to be expressed by iNKT cells. *Gzma* (granzyme a) is strongly expressed by young adult iNKT1 cells but not iNKT2 and iNKT17 cells as already reported earlier (**Figure 3G**, **Supplementary Table S1B**) (12). In contrast, aged iNKT1 cells expressed only residual levels of *Gzma*. This was confirmed by flow cytometry corroborating that predominantly CD4^−^iNKT1 cells of thymus express GZMA (**Figure 3H**). The percentage of GZMA^+^ cells decreased drastically among older CD4^−^iNKT1 cells (**Figures 3H, I**) and also the average level of GZMA per GZMA^+^ cell was much lower (**Figure 3J**). *Gzmb* mRNA was also present to a considerable extent in young adult iNKT1 cells (**Supplementary Table S1B**) yet aged cells reduced mRNA levels only moderately. When analyzing iNKT1 cells of B6 mice, we found that GZMA was expressed to a small but significant degree by young adult as well as aged cells of thymus (**Supplementary Figure S4A**). In B6 an age-related yet less pronounced control of GZMA expression was also detectable. Additional genes that exerted age-dependent regulation encompassed *Myb*, *Ezh2*, *Ccr9*, *Sox4*, and *Gas7* (**Figures 3A, C**; **Supplementary Table S1B**). Remarkably, *Sox4* was also substantially downregulated in aged iNKT17 cells.

Moreover, we observed expression of a group of genes reminiscent of a T cell precursor phenotype in young adult iNKT1 cells: *Rag1, Dntt, Ctsl, CD8a*, and *CD8b1*, respectively (**Figure 3A**; **Supplementary Table S1B**). Their expression is downregulated in aged iNKT1 cells but appears to increase among aged CD122^−^CD4^+^ iNKT2p cells. Regarding the latter we assume that a precursor-related subpopulation of iNKT cells exists that accumulate in aged iNKT2p cells (at the expense of bona fide iNKT2 cells). Whether or not one of the two identified populations, PLZF^lo^RORγt^lo^ cells (iNKT1-like), and/or PLZF^int^RORγt^+^ cells (iNKT17-like), respectively, display precursor features must await results from single cell sequencing of aged iNKT2p cells.

### iNKT17 Cells

We observed a reverse regulation of *Igf1r* and *Igfbp4*. Insulin-like growth factor 1 (IGF1) in serum is bound by a family of binding proteins regulating their activity (31). Of all existing IGF1 binding proteins, only *Igfbp4* is expressed in thymic iNKT cells of BALB/c mice. IGFBP4 blocks IGF1 activity and the inverse regulation of expression of *Igfbp4* and the receptor for IGF1, *Igf1r*, on iNKT17 cells (**Figure 4A**, **Supplementary Table S1B**) suggests a role of IGF1 for iNKT17 cell differentiation or function. Other genes expressed differently between young adult and aged iNKT17 cells encompass *Prg4*, *Pcsk1, Sox4, H2-q10*, and *Flt4*. H2-Q10 represents a class Ib MHC member that was shown to affect the development of NK and γδT cells (32). FLT-4 represents a tyrosine-protein kinase acting as a cell surface receptor for vascular endothelial growth factors. A contamination of sorted iNKT17 cells with endothelial cells can be ruled out since RNA reads for endothelial markers like *Cd34* or *Vcam1* were absent. Thus, the significance of its substantial and highly specific expression in aged iNKT17 cells remains to be explored. Interestingly however, it was found recently that *Flt4* is also prominently expressed by mucosal-associated invariant T (MAIT) cells when compared to conventional T cells (33, 34). Even though the authors did not specify the MAIT subpopulation expressing *Flt4*, MAIT17 and iNKT17 cells share functional pathways such as an IL23 driven IL17 production (35, 36) and possess an overall very similar expression profile (34).

### The Role of IL2 in Invariant Natural Killer T Cell Homeostasis

It is well established that cytokines like IL7 or IL15 impact on the homeostasis of iNKT cells (37–39). We noticed that iNKT17 cells of BALB/c thymus expressed little *Il2ra* (*Cd25*) that was significantly up-regulated particularly in aged cells (**Figure 4A**, **Supplementary Table S1B**). This was confirmed on the protein level (**Figure 4B**). Although evident, an upregulated CD25 expression was more moderate not reaching statistical significance in B6 iNKT17 cells (**Supplementary Figure S4B**). Genes coding the *Il2rb* and *Il2rg* were expressed in an age-independent manner albeit at different levels (**Supplementary Figures S4C, D**). Thus, iNKT subpopulations may be IL2 responsive to different extents either through their low affinity (*Il2rb/Il2rg*, CD122/CD132) or high affinity IL2 receptor (*Il2ra/IL2rb/Il2rg*, CD25/CD122/CD132). In thymus, T cells and DC provide IL2 in steady state (40, 41) playing a role in the homeostasis of regulatory T cells and therefore giving rise to the hypothesis that also the homeostasis of iNKT cells is influenced by IL2. Indeed, it was shown before that IL2 triggered iNKT cell proliferation [see for example (24)]. To investigate this in more detail, BALB/c iNKT cells were sorted from pooled young adult or aged thymi, labeled with a cell tracker dye and incubated *in vitro* in the presence of recombinant IL2. Cell numbers and propagation were determined 3 days later. In the following, we first focus on iNKT cells sorted from young adult thymus. In the absence of cytokine almost all cells died as expected yet we noted that iNKT17 cells were less affected by cell death (**Figure 4C**, medium only, upper left panel). In the presence of IL2, iNKT subset composition on day 3 shifted significantly compared to that determined on day 0 (**Figure 4D**). Whereas iNKT2 cells predominated in freshly isolated cells of young adult thymus, iNKT17 represented the majority in cells cultured for 3 days *in vitro* in the presence of IL2 while the frequency of iNKT1 cells was unchanged. This was reflected by cell counts (**Figure 4C**, please not the scaling of the y-axes that differ among each other). After 3 days in culture in the presence of IL2, the number of iNKT1 cells was virtually unchanged (input equals output, upper right panel). In contrast, iNKT2 cell numbers decreased and those of iNKT17 cells increased compared to input (lower panels). Cell tracker dye profiles indicated that a small subpopulation of CD4^+^iNKT1 cells underwent propagation when exposed to IL2 (representative plots **Figure 4E**, data summary ± SD underneath each plot). In contrast, the vast majority of detectable iNKT2 cells underwent several cycles of propagation yet their overall numbers rather decreased (**Figure 4E**, compare to approximately 40% of initial level iNKT2, **Figure 4C**, lower left panel) suggesting that either only a minority of them was stimulated initially or propagating cells died rather quickly under these experimental conditions. iNKT17 cells reacted quite different. A substantial proportion of cells lost a small but noticeable amount of cell tracker dye indicating a slow rate of cell division (arrow in **Figure 4E**) while only a few cells passed through more cycles of division. Remarkably, cell division observed in CD4^+^iNKT1 and iNKT2 cells correlated with a strong up-regulation of CD25 expression and a substantial increase in forward/side scatter (**Figure 4E**, **Supplementary Figure S4E**). Such phenotype is a typical characteristic of rapidly dividing regular T cells. This did not occur in non-dividing cells but also not in the bulk of slowly dividing iNKT17 cells (**Figure 4E**, **Supplementary Figure S4E**). These findings illustrate that iNKT17 cells make use of an IL2 based signaling mechanism that is absent in iNKT1 and iNKT2 cells. The observed propagation effects *in vitro* of IL2 were completely dependent on CD25 mediated signaling since in the presence of a blocking anti CD25 antibody cell divisions were suppressed largely, regardless whether they were slow or rapid in nature (**Figure 4F**). Interestingly however, IL2 promoted survival of iNKT1 cells (but not iNKT2 and iNKT17 cells) even in the presence of anti CD25 mAb (**Figure 4C**, upper right panel, lower two panels: IL2 + αCD25).

In cells of older animals we also observed a shift in subset composition (**Figure 4D**, lower panels) with iNKT17 cells predominating following 3 days of culture in the presence of IL2. Importantly, we failed to observe a fraction of rapidly dividing iNKT1 cells yet aged iNKT17 displayed the same phenotype of semi-responsiveness as young adult iNKT17 cells (**Figure 4E**, lower middle panel). Unfortunately, due to scarcity, we could not draw meaningful conclusions regarding aged iNKT2 cells upon culturing. Nevertheless, these results suggest that the capacity of IL2 to induce proliferation ceased in older iNKT1 cells whereas the mechanism of iNKT17 cell homeostasis that is based on CD25 is age independent.

Taken together, we showed that iNKT cells exerted an age-dependent phenotype. We observed considerable age-related patterns regarding expression of replication dependent histone genes and also demonstrated that aged iNKT cells of thymus propagate less well *in vivo*. We identified several candidate genes that might affect iNKT cell biology upon aging such as *Sox4*, *Ezh2*, *Tjp1*, *Gzma*, *Flt4*, *Igf1r*, and *Il2ra*. Moreover, we provided evidence that IL2 is an important factor controlling iNKT cell homeostasis particularly that of iNKT17 cells by a mechanism that depends on the high affinity IL2 receptor but is different from classical T cell activation *via* IL2.

## Discussion

Thymus represents an organ undergoing age-related involution that is accompanied by dramatic changes regarding cellularity and substructures (42). Thus, in the long run, the ongoing alteration of the micro-environment not only impacts on the generation of normal T cells (43) but also on that of iNKT cells. Indeed, it was found that the frequencies of thymic iNKT cells as well as their subset composition depend on age and also the genetic background (mouse strain) (15, 17). However, there was no clue regarding the factors causing this phenomenon and in addition, whether ageing affects the phenotype/function of iNKT cells as was observed for T cells (44). Evaluating the transcriptome data derived from young adult and aged iNKT subsets, we found that the overall expression programs of iNKT subsets were highly stable. However, the emergence of two additional iNKT2 related subpopulations among aged iNKT2 cells limited an in depth characterization of age-sensitive genes relevant for iNKT2 development and/or function. These observations demonstrated that the applied classification of iNKT subsets based on surface markers CD4/CD122 needs further refinement by adding at least one specific iNKT2 marker to address aged iNKT2 cells. Despite of considerable interest, it was beyond the scope of this study to elucidate the origin and nature of the CD122^−^CD4^+^PLZF^lo^RORγt^lo^ cells and CD122^−^CD4^+^PLZF^int^RORγt^+^ cells in further detail. This as well as a thorough investigation of other rare iNKT subsets will be addressed in future experiments and should be based on multi-color flow cytometry and single cell sequencing.

Replication-dependent histone genes comprised the largest group of genes displaying an age-dependent expression pattern. Remarkably however, regulation was not uniform but depended on the particular gene under scrutiny. More strikingly, the *in vivo* BrdU-data reflecting actual propagation of iNKT cells were in disagreement with observed expression intensities of replication-dependent histone genes: iNKT1 cells expressed these genes in general more vividly than iNKT2 cells but the latter propagated more pronounced *in vivo*. Mainly described are age-dependent posttranslational histone modifications (acetylation, methylation) (45). Yet it was also reported that expression of replication-dependent histone genes changed with age and was uncoupled from active proliferation. Thus, in retinal neurons *Hist1h1b* expression is strongest in embryonic stages while *Hist1h1c* is expressed stronger postnatally when proliferative activity ceased (46). Among all histones, histone H1 proteins differ most in amino acid composition from each other. Of the five genes coding for histone H1, we found four to be expressed strongly in iNKT cells of young adults. Three out of these four genes displayed a severe reduction in expression in aged cells (*Hist1h1a*, *Hist1h1b*, *Hist1h1d*) whereas expression of fourth, *Hist1h1c*, decreased much more moderately. This may cause a shift in the mixture of histone H1 proteins present in the older chromatin (**Supplementary Table S2A**). It was speculated that such age related changes in histone protein composition are accompanied by subtle divergences in chromatin packaging or accessibility thereby influencing gene expression (46). In contrast, replication-independent histone genes were only very moderately down-regulated in aged cells of all iNKT subtypes. In addition, the expression levels regarding iNKT subtypes were only slightly different (**Supplementary Table S2B**). This would be in line with the non-replication related functions of these histones such as control of transcription or chromatin integrity (47).

We identified a couple of genes displaying an age-regulated pattern possessing an obvious link to iNKT cell development or function such as *Sox4*, *Ezh2*, and *Gzma*. Absence of *Sox4* was shown to impair iNKT cell differentiation in general affecting most iNKT1 cells in thymus (48). We observed a significant down-modulation of *Sox4* in iNKT1 but also in iNKT17 cells thereby possibly influencing the age-dependent shift in iNKT subset composition. Along with *Sox13*, *Sox4* is also involved in development of γδT cells producing IL17 (49). There is a group of genes expressed by iNKT17 as well as by γδT cells such as *Blk*, *Scart2*, *Aqp3*, *Camk2d*, *Gpr183*, and *Sox13*, respectively, suggesting that these factors play important roles in the biology of both cell types (11, 48). We confirmed expression of these genes by iNKT17 cells but none of them was age-regulated and only *Aqp3* and *Blk* were highly specific for iNKT17 (**Supplementary Tables S1A** and **S3**). However, in the scope of investigations done here, we failed to detect an impact of BLK on iNKT17 cells. *Ezh2* was found to be down-modulated in aged iNKT cells in a subtype specific manner. EZH2 is involved in histone H3 methylation representing an important tool to control gene expression (50). It was shown that EZH2 also methylates other substrates such as PLZF thereby promoting its degradation (51). Not surprisingly, targeted deletion of *Ezh2* in T cells was shown to cause a massive expansion of iNKT cells, predominantly iNKT2 but also iNKT1 cells (51). A tuned down-regulation of *Ezh2* expression in aged iNKT cells may help explain the observation that iNKT cell frequencies as well as absolute numbers increase in thymus when mice are growing older (17).

An interesting aspect of age-regulated gene expression related to granzymes. Of the two granzyme family members expressed, *Gzma* displayed a strong age-dependency whereas *Gzmb* remained at relatively high levels also in the older iNKT1 cells. Expression of components required for alternative target cell killing like *Fasl* or *Tnfsf10* was also not age-sensitive (**Supplementary Table S3**) and killing *via* the FASL/FAS-axis was identified as a preferred mechanism engaged by iNKT cells to eliminate targets (52). Beyond their importance for cell-mediated killing, GZMA and GZMB perform additional functions. For example, GZMA can induce a potent pro-inflammatory response (53) whereas GZMB propels migration of cytotoxic lymphocytes by cleavage of basement membrane constituents (54). Based on these findings, it is tempting to speculate that upon aging the available repertoire of iNKT cells to kill targets is not restricted considerably but aging may be accompanied by a substantial loss of alternative GZMA functions. Such view is supported by the more general finding that GZMA deficient cytotoxic cells are not impaired noticeably in cell-mediated elimination of target cells (55).

We observed a variegated response of iNKT subsets upon incubation with IL2 *in vitro*. Whereas iNKT1 and iNKT2 cells isolated from young adult thymus upregulated CD25 and entered several rounds of cell division, iNKT17 cells responded with initiation of slow propagation. Strikingly, CD25 was not found to be upregulated. This was surprising considering that in iNKT17 cells *Cd25* expression in steady state was significantly more pronounced that in iNKT1 and iNKT2 cells. Nevertheless, any division activity depended on CD25 mediated signaling. Probably, IL2 responsive iNKT1 and iNKT2 cells initiate propagation like regular T cells by first signaling *via* the IL2 low affinity receptor that in turn causes upregulation of CD25 expression before cells start cycling. Why this mechanism is not triggered in iNKT17 cells under the steady state conditions mimicked in our *in vitro* assays remains enigmatic. More detailed studies will be required to decipher the underlying signaling events. At any rate, we also noted that iNKT17 cells are equipped with mechanisms supporting cell-autonomous survival *in vitro* whereas particularly iNKT2 cells are very sensitive to death without external support that may be provided by cytokines or TCR signaling (14). Of particular interest was the observation that IL2 supports survival of iNKT1 cells but not iNKT2 or iNKT17 cells even in the presence of CD25 blocking. It can be assumed that this survival mechanism depends on the low affinity receptor as only iNKT1 cells express substantial amounts of CD122. When investigating the effects of IL2 *in vitro* on aged iNKT cells, we failed to observe propagation of iNKT1 cells. However, already in young adults only a minority of iNKT1 cells divided. These responsive cells might represent iNKT1 cells that had developed recently from iNKT2 cells [see (17)] but preserved the propensity to propagate inherent to iNKT2 cells. In support of this, virtually only those iNKT1 cells divided that, like iNKT2 cells, express CD4. This would be in line with the finding that also *in vivo* significantly more BrdU-incorporation was observed in young adult CD4^+^iNKT1 cells compared to CD4^−^iNKT1 cells. In the older thymus, the reservoir of iNKT2 cells is almost exhausted wherefore also the number of CD4^+^iNKT1 cells derived recently from iNKT2 cells approximates zero. Consequently, IL2 responsive iNKT1 cells could not be detected any longer in significant amounts and also BrdU incorporation into CD4^+^iNKT1 cells dropped down to a level as observed for CD4^−^iNKT1 cells. In contrast, the iNKT17 specific response mechanism to IL2 persisted to exist also in the aged cells and thus might contribute to the age-dependent increase in iNKT17 frequencies in BALB/c thymus (17). These findings not only corroborate the efficacy of the applied markers to classify iNKT subtypes but also emphasize a thus far underrated role that may be taken by IL2 in controlling homeostasis of iNKT cells by mechanisms affecting survival and/or propagation.

In this study we found that the transcriptional patterns of iNKT cells are rather stable when comparing young adult with aged thymus. Nevertheless, we identified several genes displaying age-modulated expression. By mutually opposing and/or reinforcing effects factors like SOX4, EZH2, FLT4, and CD25 might contribute in shaping the pool of iNKT cells in aged thymus while others such as granzyme a and ZO/stathmin proteins might help to adapt the functional repertoire of iNKT cells to the needs of an aging organism. It will be interesting to explore to what extent the observed shifts in gene expression are imparted to the cells by the radically altered micro-milieu or whether they are triggered also by cell autonomous programs.

## Data Availability Statement

The datasets presented in this study can be found in online repositories. The names of the repository/repositories and accession number(s) can be found below: https://www.ncbi.nlm.nih.gov/geo/, GSE147666.

## Conflict of Interest

The authors declare that the research was conducted in the absence of any commercial or financial relationships that could be construed as a potential conflict of interest.

## Ethics statement

The animal study was reviewed and approved by Lower Saxony State Office for consumer protection and food safety (LAVES).

## Author contributions

All authors performed experiments. GB and HG designed the study. GP, GB, and HG wrote the manuscript. All authors contributed to the article and approved the submitted version.

## Funding

This work was supported by DFG grant BE1886/7-1 to GB and a DFG fellowship GE3062/1-1 to HG.
