# Supplementary material for: Impact of Aging on the Phenotype of Invariant Natural Killer T Cells in Mouse Thymus
Source: Front Immunol. 2020 Oct 30;11:575764. doi: 10.3389/fimmu.2020.575764 (PMC7662090; doi:10.3389/fimmu.2020.575764)
Supplement: Supplementary file 1 [file DataSheet_1.pdf]

Supplementary table S1A. Expression of selected signature genes in young adult and aged iNKT cells according to sequencing data (numbers of RNA reads). iNKT1=red; iNKT2=green; iNKT17=blue. *: signature genes not known so far.

Gene	Reads young adult iNKT cells			Reads aged iNKT cells		
	iNKT1	iNKT2	iNKT17	iNKT1	iNKT2p	iNKT17
Tbx21	1,579<br>1,560	80<br>100	18<br>14	2,168<br>1,920	104<br>38	15<br>19
Slamf7	1,579<br>988	0<br>0	4<br>1	1,568<br>1,672	11<br>12	0<br>0
Fgl2	6,433<br>7,365	19<br>0	2<br>2	5,995<br>5,664	4<br>18	2<br>11
Klra3	1,900<br>1,956	46<br>76	28<br>11	1,785<br>2,117	47<br>25	0<br>0
Nkg7	2,454<br>3,436	3<br>24	8<br>16	2,194<br>2,155	11<br>3	3<br>0
Ccl5	1,593<br>1,504	1<br>0	0<br>0	1,247<br>1,370	1<br>0	0<br>0
Itga1	1,748<br>1,036	2<br>0	3<br>1	3,119<br>2,895	13<br>19	2<br>0
Ly6c2	540<br>553	0<br>0	2<br>0	1,075<br>1,207	0<br>0	0<br>0
Ifng	427<br>507	8<br>8	1<br>0	305<br>322	0<br>0	1<br>0
Il6ra	1<br>0	376<br>615	68<br>148	1<br>3	1,427<br>1,505	91<br>82
Ccr7	25<br>100	654<br>611	124<br>165	9<br>0	461<br>444	32<br>75
Il4	10<br>44	263<br>307	75<br>82	6<br>6	116<br>136	49<br>68
*Nrp2**	72<br>59	578<br>622	31<br>18	100<br>143	899<br>1,171	0<br>4
Slamf6	167<br>157	1,016<br>999	388<br>382	96<br>79	648<br>689	95<br>109
Sdc1	0<br>0	75<br>78	3,542<br>3,379	4<br>0	532<br>540	4,508<br>5,057
Rorc	223<br>118	73<br>81	2,834<br>2,579	23<br>60	478<br>454	2,851<br>2,770
Il23r	0<br>0	77<br>72	4,004<br>3,574	0<br>0	480<br>607	4,889<br>5,159
Blk	6<br>0	85<br>65	6,079<br>5,063	1<br>0	504<br>526	6,623<br>7,185
Il1r1	2<br>0	77<br>87	3,745<br>3,280	0<br>0	367<br>534	3,423<br>3,183
Mmp25	0<br>0	9<br>63	2,212<br>2,351	1<br>0	403<br>422	3,513<br>3,927
Socs3	94<br>223	112<br>82	1,196<br>1,287	140<br>115	193<br>240	1,276<br>1,265
Coro2b	4<br>0	58<br>142	1,197<br>1,521	2<br>1	220<br>319	1,724<br>1,953
Stab2	1<br>0	7<br>0	1,254<br>931	0<br>0	51<br>84	1,078<br>1,089
Serpinb1a	5<br>4	5<br>0	741<br>429	4<br>1	87<br>135	869<br>975
Ascl1	0<br>0	16<br>15	852<br>792	0<br>0	94<br>52	619<br>766
*Ankrd63**	5<br>11	182<br>250	1,210<br>1,388	1<br>3	215<br>151	1,214<br>1,045

Supplementary table S1B. Expression of selected genes differently expressed in young adult versus aged iNKT cells according to sequencing data (numbers of RNA reads). iNKT1=red; iNKT2=green; iNKT17=blue: Numbers>50 reads are colored accordingly when a regulation (ratio reads) >4 between young adult and aged cells was observed. Genes mentioned in the text are in bold. Genes in red shaded box: Genes possibly related to precursor iNKT cells that display down-regulated expression in aged iNKT1 cells.

Gene	Reads young adult iNKT cells			Reads aged iNKT cells		
	iNKT1	iNKT2	iNKT17	iNKT1	iNKT2p	iNKT17
Gzma	4,106<br>4,140	11<br>0	6<br>17	297<br>265	1<br>5	2<br>1
Gzmb	2,842<br>2,421	5<br>0	21<br>28	1,016<br>1,008	0<br>0	3<br>0
Myb	1,267<br>949	1,693<br>1,660	2,217<br>1,942	161<br>106	1,454<br>1,534	1,097<br>817
Ezh2	837<br>583	397<br>239	313<br>256	99<br>151	299<br>276	88<br>85
Stmn1	789<br>600	496<br>451	285<br>249	108<br>75	220<br>187	39<br>36
Ccr9	587<br>640	427<br>330	76<br>128	94<br>94	703<br>723	9<br>31
Sox4	502<br>313	494<br>657	589<br>550	67<br>59	574<br>632	75<br>99
Tjp1	128<br>109	0<br>5	11<br>11	1,004<br>1,058	4<br>2	7<br>27
Gas7	153<br>80	0<br>0	0<br>1	516<br>534	2<br>8	0<br>0
Dntt	1,708<br>1,854	171<br>174	124<br>256	124<br>170	696<br>604	17<br>41
Cd8b1	1,350<br>1,012	184<br>264	114<br>238	156<br>197	843<br>915	32<br>105
Ctsl	1,742<br>1,282	241<br>228	171<br>217	157<br>145	689<br>746	49<br>80
Themis	981<br>999	541<br>452	117<br>154	108<br>136	889<br>1,182	22<br>30
S1pr1	102<br>113	459<br>261	68<br>57	233<br>242	98<br>162	10<br>5
Igf1r	33<br>2	160<br>342	2,647<br>3,552	73<br>62	2,440<br>3,025	6,483<br>6,629
Rad50	280<br>137	133<br>179	304<br>401	513<br>507	625<br>639	803<br>711
Prg4	10<br>0	312<br>487	73<br>93	93<br>82	1,688<br>1,296	379<br>490
Pcsk1	9<br>0	416<br>260	459<br>523	0<br>3	25<br>78	43<br>48
Igfbp4	130<br>148	2,072<br>1,280	810<br>638	7<br>6	458<br>712	26<br>34
Flt4	0<br>0	0<br>0	88<br>95	1<br>0	66<br>54	1,008<br>954
Il2ra	195<br>48	14<br>23	346<br>391	88<br>85	281<br>275	1,842<br>1,871

Supplementary table S2A. Expression of replication-dependent histone genes in young and aged iNKT cells. Given are the read numbers for the 15 most highly expressed histone genes in young adult BALB/c mice. The degree of down regulation in aged iNKT subtypes was determined by the ratios of values obtained for the corresponding population. For example for *Hist1h1c* expression by iNKT1 cells: 1y/o = (17,084+19016) : (5442+5172) = 3,40. Genes depicted in red: the degree of downregulation is high for iNKT1 (>14) and higher than average also for iNKT17 (>5). For the latter the degree of down regulation is rather uniform: 2,27 ± 0,48 (±SD).

Gene	Reads young adult iNKT cells			Reads aged iNKT cells (+ ratio regulation)					
	iNKT1	iNKT2	iNKT17	iNKT1	1y/o	iNKT2p	2y/o	iNKT17	17y/o
Hist1h1c	17,084<br>19,016	9,744<br>5,022	7,386<br>4,939	5,442<br>5,172	3,40	4,029<br>4,093	1,82	4,047<br>3,834	1,56
Hist1h1d	8,927<br>12,178	4,752<br>2,524	2,305<br>1,832	674<br>776	14,6	1,513<br>1,380	2,52	350<br>460	5,11
Hist2h2bb	8,082<br>11,692	3,932<br>2,295	3,234<br>2,457	1,533<br>1,937	5,70	1,224<br>1,252	2,51	1,733<br>2,025	1,51
Hist1h1e	5,095<br>4,223	3,193<br>2,334	2,581<br>2,116	1,409<br>1,403	3,31	1,334<br>1,206	2,18	1,146<br>1,098	2,09
Hist1h4d	4,143<br>6,971	2,927<br>2,155	1,524<br>1,686	1,391<br>1,303	4,13	1,053<br>839	2,69	913<br>696	2,00
Hist2h2ac	3,688<br>6,231	2,327<br>1,290	1,631<br>1,208	849<br>789	6,06	839<br>686	2,37	1,024<br>779	1,57
Hist1h1b	3,353<br>4,234	1,084<br>428	418<br>391	86<br>98	41,2	343<br>240	2,59	19<br>35	15,0
Hist1h1a	3,337<br>4,475	803<br>464	409<br>508	112<br>112	34,9	262<br>317	2,19	30<br>92	7,52
Hist1h2be	2,617<br>3,629	1,193<br>620	423<br>484	540<br>615	5,41	456<br>427	2,05	207<br>310	1,75
Hist1h2bn	2,347<br>2,990	589<br>326	263<br>233	68<br>86	34,7	175<br>261	2,10	13<br>69	6,05
Hist4h4	2,228<br>3,886	2,495<br>1,646	1,409<br>1,129	888<br>765	3,70	763<br>601	3,04	756<br>483	2,05
Hist1h2ak	2,223<br>2,400	623<br>450	292<br>362	132<br>127	17,9	301<br>372	1,59	54<br>112	3,94
Hist1h2af	2,090<br>2,500	416<br>284	214<br>217	67<br>85	30,2	239<br>311	1,27	25<br>21	9,37
Hist1h3b	1,535<br>2,324	424<br>222	168<br>191	52<br>68	32,2	145<br>155	2,15	10<br>27	9,70
Hist1h4h	1,439<br>2,021	1,014<br>755	569<br>615	330<br>295	5,54	324<br>266	3,00	269<br>261	2,23

Supplementary Table S2B. Expression of replication-independent histone genes in young and aged iNKT cells. Setup of the table as in S2A.

Gene	Reads young adult iNKT cells			Reads aged iNKT cells (+ ratio regulation)					
	iNKT1	iNKT2	iNKT17	iNKT1	1y/o	iNKT2p	2y/o	iNKT17	17y/o
H2afx	1,818<br>1,656	693<br>524	437<br>420	284<br>242	6,60	388<br>292	1,79	211<br>189	2,14
H2afy	1,688<br>970	1,096<br>1,159	1,263<br>1,025	1,310<br>1,282	1,02	1,026<br>1,011	1,11	820<br>727	1,48
H2afz	1,145<br>1,355	747<br>448	708<br>574	573<br>519	2,29	383<br>396	1,53	484<br>361	1,52
H2afj	742<br>1,238	816<br>796	542<br>462	624<br>552	1,68	431<br>498	1,74	405<br>339	1,35
H2afv	452<br>361	305<br>303	279<br>331	219<br>256	1,71	203<br>181	1,58	242<br>261	1,21
H3f3b	2,899<br>3,433	2,741<br>2,030	2,391<br>1,849	1,597<br>1,789	1,87	1,565<br>2,091	1,30	1,328<br>1,486	1,51
H3f3a	312<br>524	575<br>408	422<br>410	256<br>273	1,58	298<br>295	1,66	312<br>237	1,52

Supplementary table S3. Expression of selected genes in young adult and aged iNKT cells according to sequencing data (RNA reads). Data source as in Fig. 2.

Gene	Reads young adult iNKT cells			Reads aged iNKT cells		
	iNKT1	iNKT2	iNKT17	iNKT1	iNKT2p	iNKT17
Scart2[1]	186<br>135	602<br>685	4,665<br>3,741	149<br>125	625<br>846	5,247<br>4,909
Aqp3	104<br>128	58<br>65	1,389<br>1,090	100<br>111	179<br>281	1,595<br>1,592
Camk2d	289<br>298	148<br>159	234<br>251	341<br>381	234<br>306	269<br>277
Gpr183	1,227<br>1,741	437<br>418	1,435<br>1,187	1,547<br>1,765	569<br>802	1,677<br>1,644
Sox13	16<br>4	532<br>1,100	1,260<br>2,583	13<br>15	1,483<br>1,268	2,766<br>3,074
Fasl	869<br>1,092	36<br>20	9<br>7	884<br>878	34<br>25	7<br>35
Tnfsf10	1,776<br>1,281	413<br>410	244<br>249	1,363<br>1,361	389<br>363	263<br>278

1) Scart2 = 5830411N06Rik

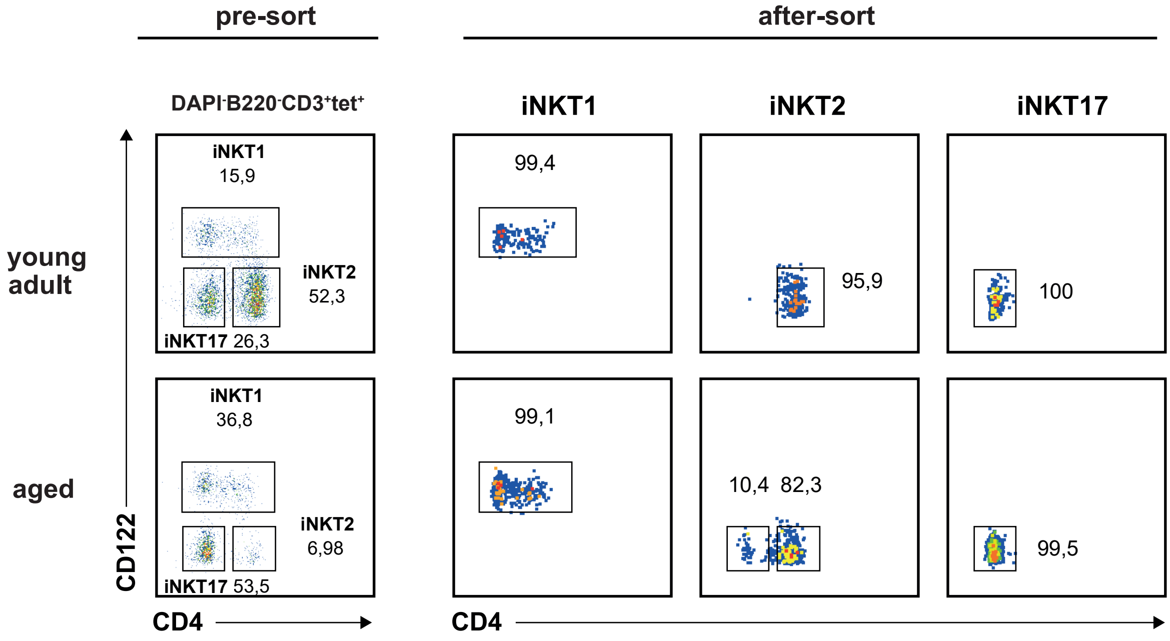

Supplementary Figure 1. Sorting strategy of iNKT subsets from young adult and aged mice. The plots illustrate the gating strategy (pre-sort) for live cell sorts (one out of two performed) to obtain iNKT subpopulations as also shown in Fig. 1B but using a flow cytometry sorter. In each experiment thymic cells were pooled from either 3 young adult (upper panel) or 3 aged (lower panel) BALB/c mice. After-sort: Small aliquots of sorted subsets were re-analyzed to check for purity that is indicated by the numbers accompanying the gates. Note that in aged iNKT2 cells an impurity of approximately 10% iNKT17 cells was found.

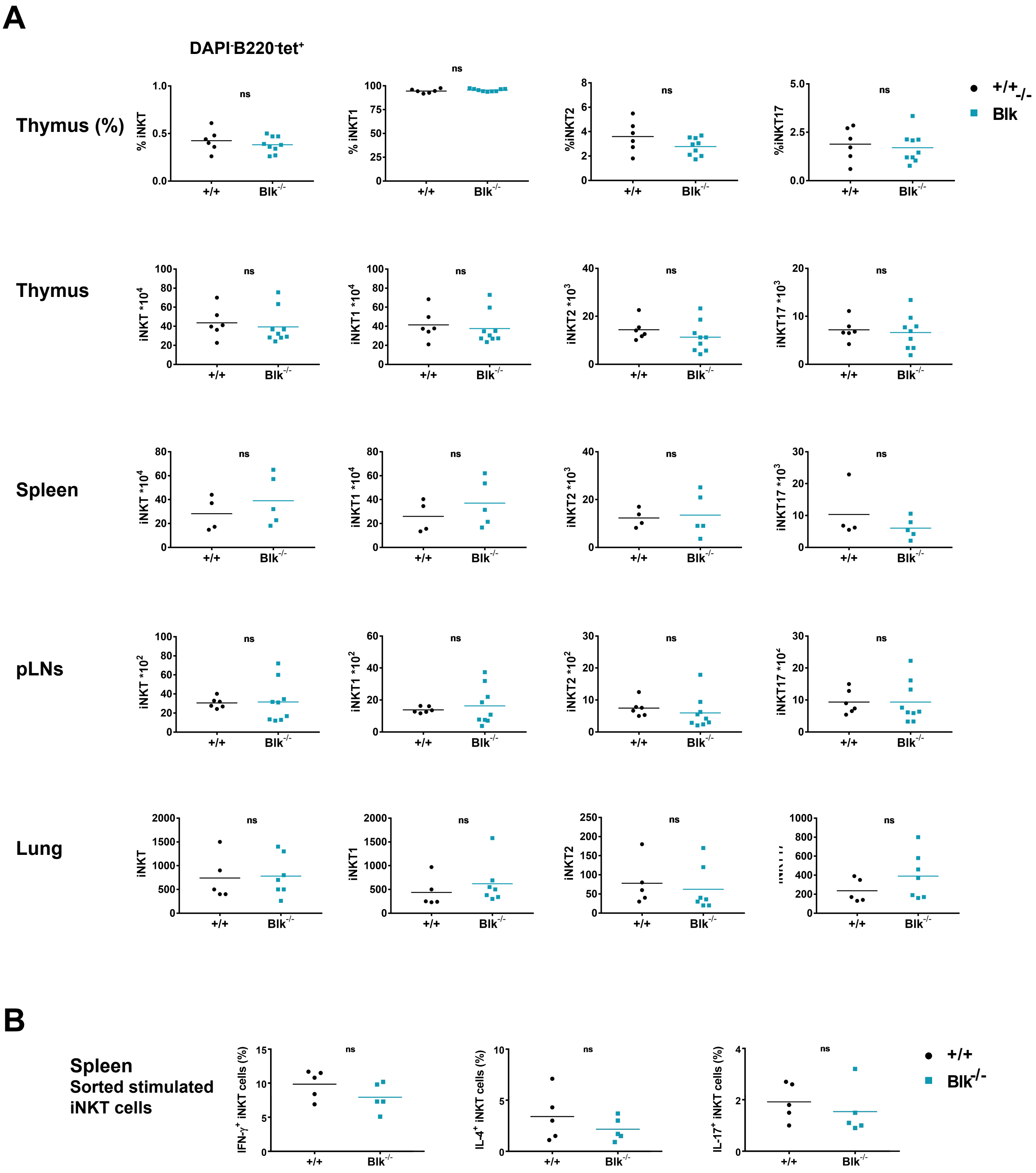

Supplementary Figure 2. *Blk* deficiency does not have an impact on iNKT cell frequencies and cytokine production. (A) iNKT cell frequencies and cell numbers in BL6 wt and *Blk-/-* mice as determined by flow cytometry. Gating as shown in fig. 1B. Data were summarized from at least 3 independent experiments. Each dot represents data from one animal. (**B)** IFN-γ, IL-4 and IL-17 production of iNKT cells from wt and *Blk-/-* BL6. Total splenic iNKT cells were sorted and stimulated with anti-CD3 and anti-CD28 for 16h as described in Experimental Procedures. Data are collected from 5 independent experiments (dots) and statistically evaluated with unpaired t test. ns: not significant.

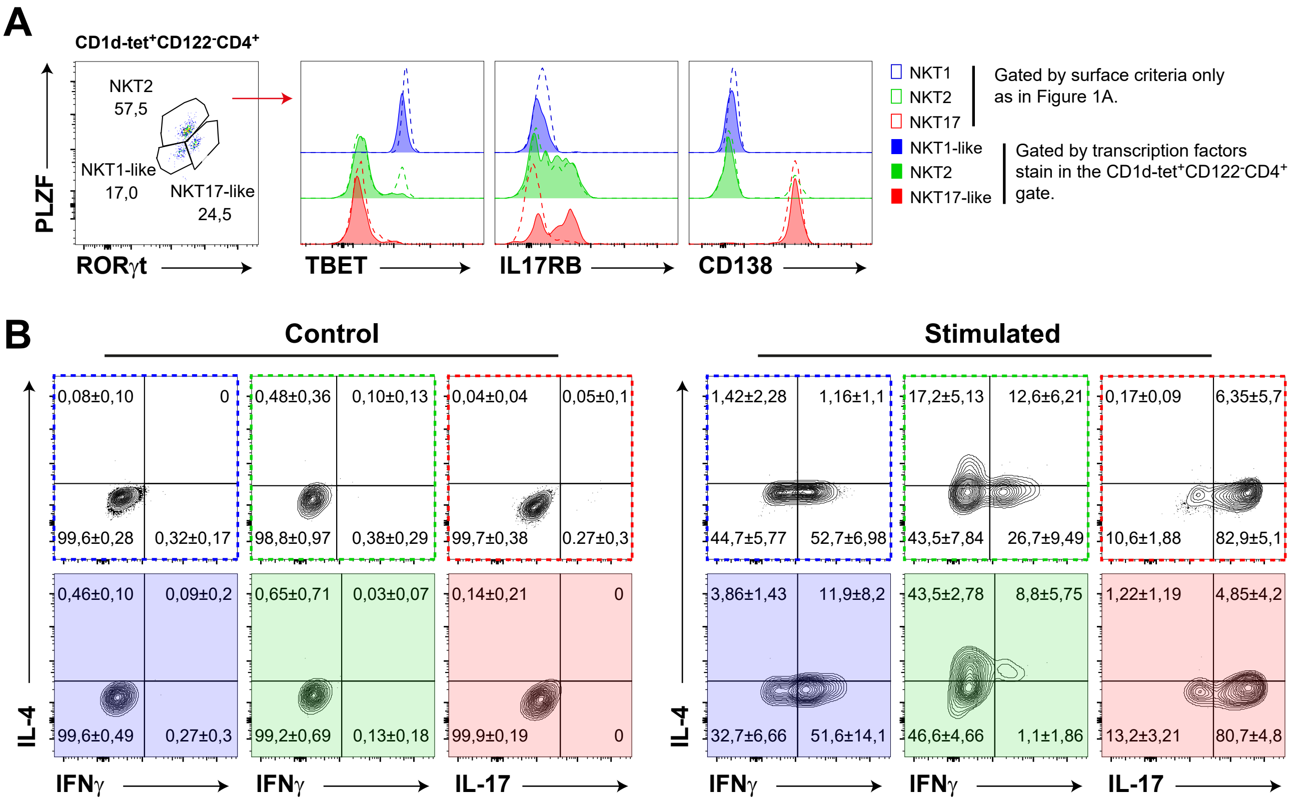

Supplementary Figure 3. Characterization of aged iNKT2p cells. (A) Thymi from 35 week old female BALB/c mice were investigated by flow cytometry. Gating for surface markers was done as shown in Figure 1A. The representative plot (left panel) depicts the three main subsets in aged iNKT2p cells based on additional stain for PLZF and RORγt (see also Figure 1B). The histograms (middle part) show the expression of TBET, IL17RB and CD138 (syndecan) by either iNKT1 ($CD122^{+}CD4^{+/-}$), iNKT2 ($CD122^{-}CD4^{+}$) and iNKT17 ($CD122^{-}CD4^{-}$) cells based only on gating by surface markers (dashed lines) or by the subsets of aged iNKT2p cells in the $CD122^{-}CD4^{+}$ gate but now gated additionally according to their expression of PLZF and ROR (filled and colored). **(B)** Production of cytokines as indicated following PMA/ionomycin stimulation. Open boxes (framed by dashed lines) correspond to the cytokine production profile of cells gated by surface markers only (as in A) or by the cells dissected further into the iNKT2p subpopulations as defined by PLZF/RORγt expression. Data are shown for 5 mice (thymi) analyzed (mean±SD).

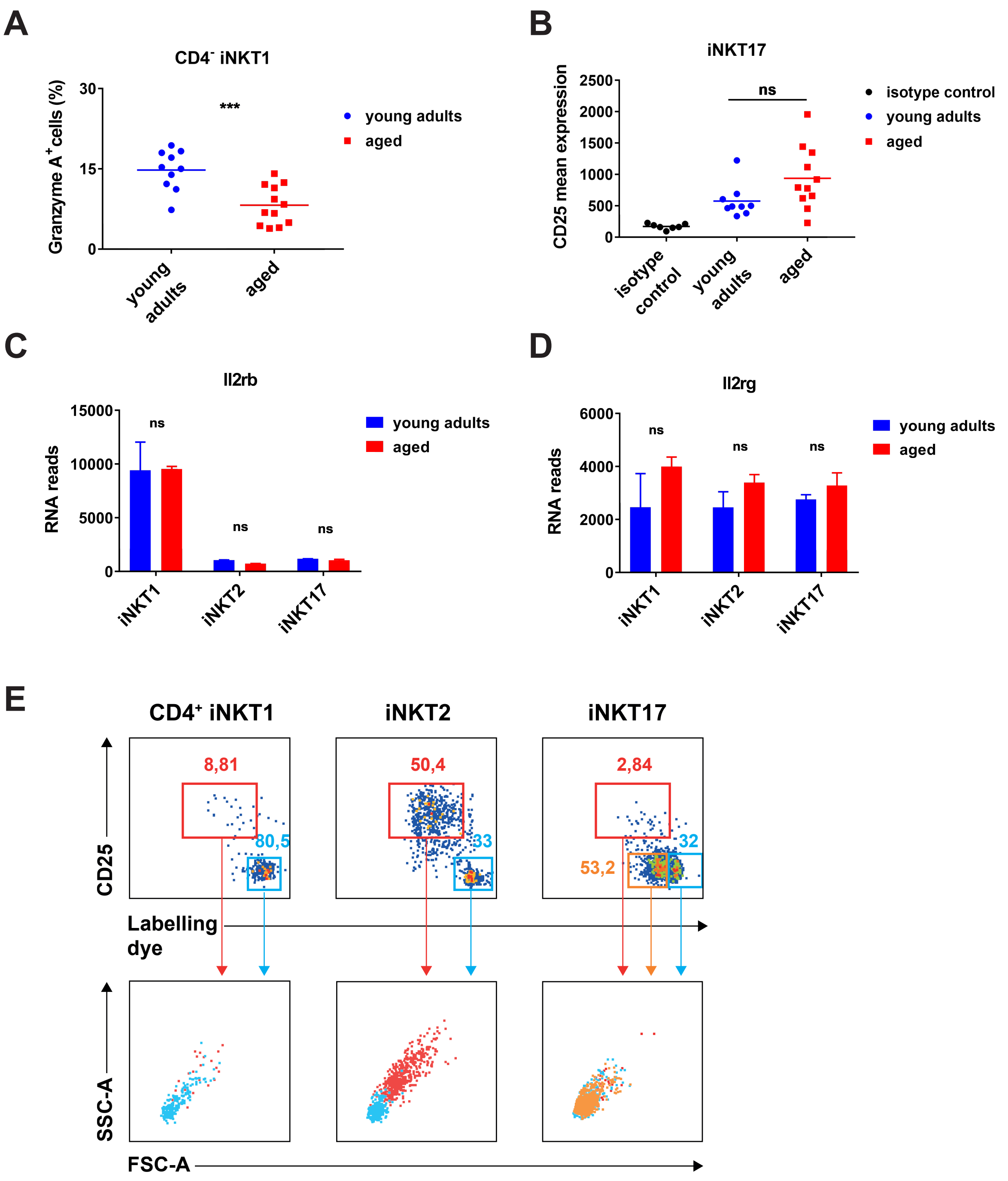

Supplementary Figure 4. (A) Granzyme A+ cell frequencies among thymic CD4⁻iNKT1 cells from young adult and aged BL6 mice using the same analytic strategy as for BALB/c cells (Fig. 3I). Data are collected from 5 independent experiments. Each dot represents data from one animal. **(B)** Mean expression of CD25 in thymic iNKT17 cells from young adult and aged BL6 mice using the same gating strategy as for BALB/c cells (Fig. 4B). Data are collected from 5 independent experiments. Each dot represents data from one animal. **(C-D)** *Il2rb* and *Il2rg* RNA reads (mean ± sd), same source as in Fig.2. **(E)** Alternative presentation of levels of CD25 and cell tracker dye (upper panel) in the iNKT cell subsets displayed in Fig 4E as well as forward/side scatter of the color-gated populations (lower panel) on day 3 after stimulation with IL2. Unpaired t test was performed in **(A)**. One-way ANOVA followed by Tukey's multiple comparisons test were performed in **(B)**. Two-way ANOVA followed by Sidak's multiple comparisons test were performed in **(C)** and **(D)**. ***p <0.001, ns=not significant.
